# A rare case of abdominal aortic coarctation in a pediatric patient

**DOI:** 10.1097/RC9.0000000000000475

**Published:** 2026-05-29

**Authors:** Javad Salimi, Saeed Soltani, Amir Shokri, Hossein Ghayyemi, Siamak Mousazade, Mohammadreza Zafarghandi

**Affiliations:** a Department of Vascular Surgery, Sina Hospital, Tehran University of Medical Sciences, Tehran, Iran; b Department of Vascular Surgery, Faculty of Medicine, Tehran University of Medical Sciences, Tehran, Iran

**Keywords:** abdominal aortic coarctation, aortic bypass, case report, hypertension, nephrectomy, pediatric vasculitis

## Abstract

**Introduction:**

Abdominal aortic coarctation (AAC) is a rare cause of renovascular hypertension in pediatric patients, particularly when linked to vasculitis. It presents unique diagnostic and therapeutic challenges. It is often associated with severe hypertension and lower limb ischemia. This case underscores the crucial role of timely surgical intervention in complex vascular cases.

**Case presentation:**

We report a pediatric patient with severe hypertension and diminished renal function. Imaging revealed segmental abdominal aortic narrowing with left renal artery stenosis. Medical management and attempted angioplasty failed. Aortic bypass grafting was performed, along with left nephrectomy due to irreversible ischemic damage. The patient showed marked postoperative improvement in blood pressure and end-organ perfusion.

**Clinical discussion:**

AAC with an inflammatory component often resists conservative and endovascular therapies due to the development of fibrotic changes in the vessel wall. Surgical revascularization provides durable outcomes and improved control of hypertension. Identification of vasculitis necessitates long-term immunologic monitoring and treatment.

**Conclusion:**

Surgical bypass is the preferred intervention in pediatric AAC refractory to medical and endovascular management, primarily when associated with vasculitis. Early multidisciplinary involvement is essential to prevent permanent organ damage and optimize outcomes.

## Introduction

Abdominal aortic coarctation (AAC), also referred to as mid-aortic syndrome, is a rare vascular condition characterized by narrowing of the abdominal aorta, typically affecting the segment between the distal thoracic aorta and the aortic bifurcation^[^1,2^]^. This narrowing may also involve the renal and visceral arteries, leading to end-organ hypoperfusion and renovascular hypertension^[^3^]^. AAC most commonly presents in children and young adults with symptoms such as refractory hypertension, claudication, or failure to thrive^[^3–6^]^. While the etiology may be congenital – due to improper fusion of the embryonic dorsal aorta – it can also be acquired in association with vasculitis, fibromuscular dysplasia, or neurofibromatosis^[^7,8^]^. Diagnosis relies heavily on imaging techniques, such as computed tomography angiography, magnetic resonance angiography, and Doppler ultrasonography ^[^9–11^]^ Recent advances in patient-specific blood flow modeling have further enhanced the utility of imaging in surgical decision-making^[^12^]^. Management strategies range from antihypertensive therapy and endovascular intervention to definitive surgical bypass, depending on anatomical complexity and symptom severity^[^13,14^]^. In this report, we describe open surgical management in a child with refractory AAC, with emphasis on clinical presentation, imaging, and surgical outcome. This work has been reported in line with the SCARE criteria (2025 update)^[^15^]^.

HIGHLIGHTS

- Adult aortic coarctation is underrecognized and can present with limb ischemia and hypertension.
- Inflammatory etiologies (e.g., Takayasu arteritis) drive fibrosis and complicate revascularization.
- Endovascular therapy in active vasculitis has higher restenosis – patient selection is critical.
- Open reconstruction remains durable for complex disease (≈90% patency at 5 years).
- Long-term control depends on immunosuppression, plus structured imaging surveillance.
- Recurrence/restenosis occurs in ~10–20%; contemporary 5-year survival exceeds 95%.
- Early diagnosis and multidisciplinary care can prevent irreversible renal and vascular injury.

## Case presentation

**Figure 1.**
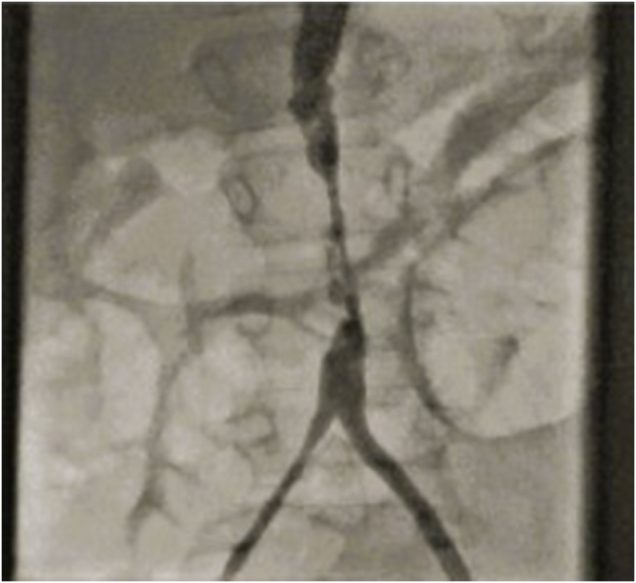
Catheter angiographic examination demonstrates significant narrowing of the abdominal aorta in its mid-segment, characteristic of coarctation. Multiple collateral vessels are visible, indicating chronic compromise of distal perfusion.

**Figure 2.**
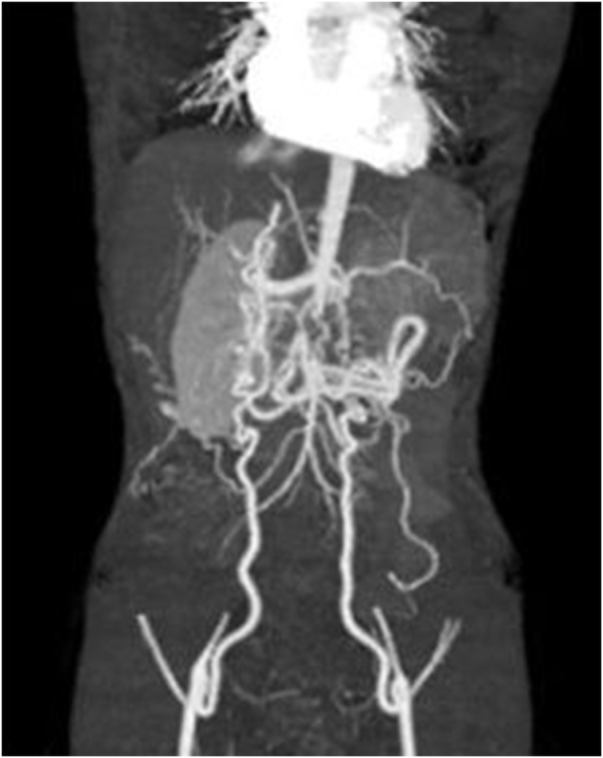
Initial computed tomography angiography of the patient. Long-segment coarctation of the abdominal aorta is demonstrated extending from below the renal arteries to just above the aortic bifurcation. Marked stenosis of the left renal artery is evident, with the left kidney appearing atrophic and poorly perfused. Extensive collateral circulation is visualized, suggesting a chronic and critical compromise of distal aortic flow, consistent with severe mid-aortic syndrome.

**Figure 3.**
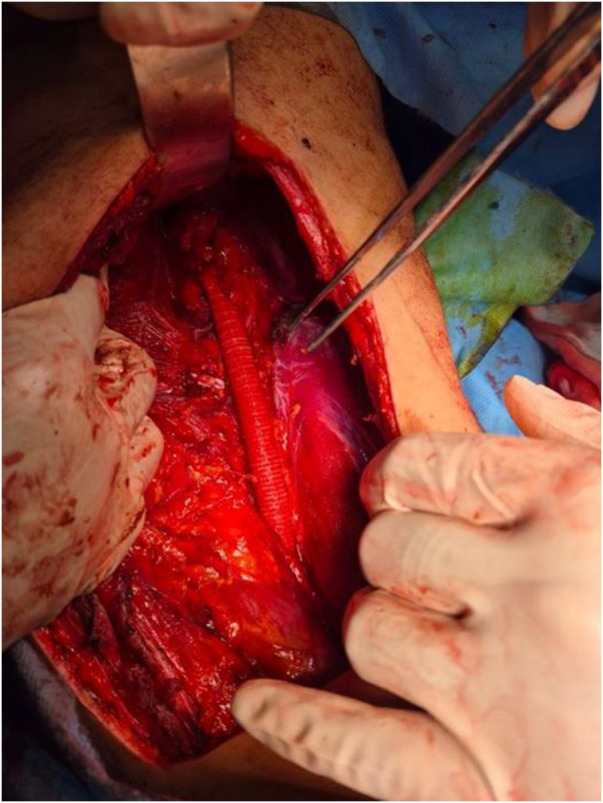
Intraoperative photograph showing placement of a 12-mm Dacron graft bypassing the coarcted segment of the abdominal aorta.

A 12-year-old girl presented with a 3-year history of drug-resistant hypertension, poorly responsive to multidrug regimens, including calcium channel blockers, beta-blockers, and angiotensin-converting enzyme inhibitors. She also reported progressive bilateral lower limb claudication, difficulty walking long distances, and fatigue with mild exertion. Her family reported frequent early morning headaches, dizziness, and occasional blurred vision, suggestive of end-organ effects of uncontrolled hypertension. On physical examination, her upper limb blood pressure measured 165/105 mmHg, while lower extremity pressures were significantly reduced, yielding a systolic pressure gradient exceeding 50 mmHg. Bilateral femoral pulses were diminished and delayed, accompanied by lower limb muscle wasting and coolness to the touch. Evaluation by pediatric cardiology and nephrology specialists had ruled out other common causes of secondary hypertension, including coarctation of the thoracic aorta, renal parenchymal disease, and endocrine etiologies. Three years earlier, AAC had been diagnosed via angiography at an outside facility (Fig. 1). Repeated CT angiography at our center demonstrated a long-segment coarctation distal to the renal arteries, extending to 2 cm above the aortic bifurcation, with critically impaired distal perfusion sustained by collateral circulation (Fig. 2). Significant stenosis was noted in the celiac trunk and renal arteries, with near-total occlusion of the left renal artery and absent contrast enhancement. The left kidney appeared severely atrophic, and a nuclear renal scan confirmed non-function. One year prior to presentation, two unsuccessful percutaneous transluminal angioplasty attempts had been made elsewhere, which failed due to technical challenges and yielded no hemodynamic improvement. Given the refractory nature of her condition and progressive end-organ damage, surgical repair was pursued. Advanced imaging and planning CT angiography DICOM files were processed using 3D Slicer to perform segmentation and generate a 3D model of the abdominal vasculature. This allowed for detailed visualization of the coarctation and assessment of collateral flow. The surgical team used this information to determine the graft path and plan for simultaneous nephrectomy. The surgical procedure performed was an extra-anatomic bypass graft for complex aortic coarctation, utilizing a retroperitoneal approach through the eighth intercostal space. This specific incision location creates thoracoabdominal exposure, allowing simultaneous access to the lower thoracic aorta and the upper abdominal aorta/aortic bifurcation. Following extensive multidisciplinary preoperative optimization involving anesthesia, cardiology, and nephrology, this complex surgery successfully bypassed a severe aortic coarctation using a Dacron graft from the descending thoracic aorta to the bifurcation and removed a non-functioning left kidney (Fig. 3). Postoperatively, femoral pulses were immediately palpable, and the patient’s blood pressure normalized without the need for antihypertensive therapy. Renal function remained stable throughout the admission. The patient was monitored in the intensive care unit for 48 hours and then transferred to the surgical ward, with progressive ambulation and advancement of diet as tolerated. Serum creatine kinase levels and electrolytes were checked serially and remained within reference ranges, and there was no clinical or laboratory evidence of rhabdomyolysis. The patient was discharged home on postoperative day 10 in good condition. At the 3-month follow-up, the patient remained normotensive off medication, with complete resolution of claudication and exertional fatigue, and no graft-related complications on clinical assessment.

## Discussion

Coarctation or segmental narrowing of the aorta in adults arises from heterogeneous etiologies that include degenerative atherosclerotic disease, postsurgical or post-inflammatory scarring, and vasculitis such as Takayasu arteritis. Inflammatory arteriopathy is characterized by transmural inflammation, concentric fibrosis, and diffuse arterial involvement^[^16,17^]^, features that complicate endovascular therapy and influence long-term outcomes. Accordingly, management must be individualized, integrating anatomic suitability, disease activity, and the likelihood of durable patency. In suspected Takayasu arteritis, classification follows established criteria, including the Sharma and ACR 1990 criteria^[^18,19^]^. Endovascular approaches (balloon angioplasty ± stenting) are attractive for focal, noninflammatory lesions; however, in inflammatory aortitis, these strategies are limited by rigid, fibrotic walls and a propensity for recoil and neointimal hyperplasia, translating into higher restenosis and reintervention rates. In contrast, open surgical revascularization – whether *in situ* or extra-anatomic bypass – remains an effective option in selected patients with extensive disease, heavy calcification, or inflammation^[^20^]^. Reported primary patency after open reconstruction approaches approximately 90% at 5 years, supporting surgery as a durable modality when endovascular outcomes are expected to be suboptimal^[^21,22,23^]^. Renal involvement is common in proximal aortic disease due to chronic renovascular compromise. When a kidney is rendered nonfunctional with irreversible parenchymal loss, nephrectomy may be appropriate to address refractory hypertension, recurrent infection, or pain, and to simplify postoperative management. This decision should be anchored in objective assessments of split renal function and the feasibility of renal revascularization. Long-term care hinges on controlling the underlying disease process and preserving conduit patency. In inflammatory etiologies, immunosuppressive therapy (e.g., glucocorticoids with or without steroid-sparing agents) is central to induce and maintain remission, reduce restenosis, and protect downstream vascular beds^[^24^]^. Lifelong surveillance is required given reported recurrence or restenosis in roughly 10–20% of patients; follow-up typically integrates clinical assessment, noninvasive hemodynamic testing (e.g., ABI), and periodic vascular imaging tailored to the repair type and disease activity. With contemporary multidisciplinary management, 5-year survival exceeds 95%. Our case aligns with these principles: the choice of open bypass in the setting of extensive disease produced immediate hemodynamic improvement and early durability, while nephrectomy addressed irreversible renal injury. The experience underscores that a strategy prioritizing disease control, durable revascularization, and structured surveillance can yield favorable intermediate outcomes, particularly in patients for whom endovascular therapy is disadvantaged by active inflammation and fibrosis.

Rhabdomyolysis risk: Major thoracoabdominal surgery, especially involving prolonged muscle retraction and potential ischemia/reperfusion, carries a significant risk of rhabdomyolysis (muscle breakdown releasing myoglobin, potentially causing kidney damage). Its absence indicates careful surgical technique, adequate perfusion, and likely appropriate intraoperative positioning and padding.

Electrolyte disturbance risk: Major vascular surgery, significant fluid shifts, blood loss, and concurrent nephrectomy all create a high risk of electrolyte imbalances (e.g., sodium, potassium, calcium abnormalities). The absence of such disturbances reflects meticulous intraoperative fluid management, hemodynamic stability, and likely well-preserved function, as discussed in the remaining sections.

Furthermore, detecting an underlying inflammatory cause carries long-term consequences, necessitating ongoing rheumatologic assessment and immunologic workup. This case reinforces the importance of tailored treatment plans – frequently requiring a multidisciplinary collaboration – for managing rare and intricate vascular disorders. Vascular patients require individualized management, particularly when medical and endovascular strategies fail. This case demonstrates the importance of thorough anatomical assessment and timely surgical planning. Nephrectomy was necessary given irreversible renal ischemia. Multidisciplinary collaboration is essential to optimize outcomes in complex pediatric vascular surgery. Histopathology showed evidence of chronic transmural inflammation, segmental intimal thickening, and lymphoplasmacytic infiltration, suggestive of a vasculitis process.

## Conclusion

In summary, AAC in pediatric patients, particularly when linked to vasculitis etiologies, requires prompt recognition, a multidisciplinary approach, and a tailored treatment plan. While surgical revascularization is invasive, it remains the definitive therapy in cases where medical and endovascular management have failed, offering the best chance for recovery and long-term stability^[^25^]^. Histopathological assessment remains crucial for guiding postoperative management and long-term surveillance.

## Data Availability

The data that support the findings of this study are available from the corresponding author upon reasonable request.
